# Quantum Iterative Deepening with an Application to the Halting Problem

**DOI:** 10.1371/journal.pone.0057309

**Published:** 2013-03-08

**Authors:** Luís Tarrataca, Andreas Wichert

**Affiliations:** Department of Informatics, INESC-ID/Instituto Superior Técnico, Lisbon, Portugal.; University of Nottingham, United Kingdom

## Abstract

Classical models of computation traditionally resort to halting schemes in order to enquire about the state of a computation. In such schemes, a computational process is responsible for signaling an end of a calculation by setting a halt bit, which needs to be systematically checked by an observer. The capacity of quantum computational models to operate on a superposition of states requires an alternative approach. From a quantum perspective, any measurement of an equivalent halt qubit would have the potential to inherently interfere with the computation by provoking a random collapse amongst the states. This issue is exacerbated by undecidable problems such as the *Entscheidungsproblem* which require universal computational models, *e.g.* the classical Turing machine, to be able to proceed indefinitely. In this work we present an alternative view of quantum computation based on production system theory in conjunction with Grover's amplitude amplification scheme that allows for (1) a detection of halt states without interfering with the final result of a computation; (2) the possibility of non-terminating computation and (3) an inherent speedup to occur during computations susceptible of parallelization. We discuss how such a strategy can be employed in order to simulate classical Turing machines.

## Introduction

Classically, the status of any computation can be determined through a halt state. The concept of the halting state has some important subtleties in the context of quantum computation. The first one of these relates to quantum state evolution which needs to be expressed through unitary operators that represent reversible mappings. As a consequence, two successive states cannot be equal. Ekert draws attention to this fact stating that there are two possibilities to circumvent such an issue, namely [1]: either run the computation for some predetermined number of steps or alternatively employ a halt flag. This flag is then employed by a computational model to signal an end of the calculation. Traditionally, such a flag is represented by a halt bit which is initialized to 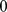 and set to 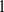 once the computation terminates. Accordingly, determining if a computation has finished is simply a matter of checking if the halt bit is set to 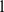, a task that can be accomplished through some form of periodic observation.

Furthermore, undecidable problems, such as the famous *Entscheidungsproblem* challenge proposed by Hilbert in [2], require that computational models be capable of proceeding indefinitely, a procedure that can only be verified through a recurrent observation of a halt bit. Classical models of computation are able to execute undecidable problems since their formulation allows for the use of such a flag without affecting the overall result of the calculation. Undecidable problems are important because they demonstrate the existence of a class of problems that does not admit an algorithmic solution no matter how much time or spatial resources are provided [3]. This result was first demonstrated by Church [4] and shortly after by Turing [5].

### Problem

Deutsch [6] was the first to suggest and employ such a strategy in order to describe a quantum equivalent of the Turing machine which employs a compound system 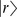 expressed as a tensor of two terms, *i.e.* 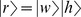, spanning a Hilbert space 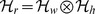. The component 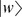 represents a work register of unspecified length and 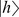 a halt qubit which is used in an analogous fashion to its classical counterpart. However, Deutsch's strategy turned out to be flawed, namely suppose a unitary computational procedure 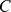 acting on input 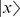 is applied 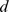 times and let 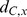 represent the number of steps required for a procedure 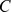 to terminate on input 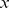. Then it may be possible that there exist 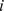 and 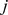 for which 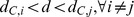. Now, lets consider what happens when we are in the presence of such a behaviour and 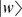 is initialized as a superposition of the computational basis. Then those states which only require a number of computational steps less than or equal to 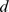 in order to terminate will have the halt qubit set to 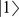, whilst the remaining states will have the same qubit set to 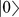. This behaviour effectively results in the overall superposition state 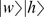 becoming entangled as exemplified by Expression 1, where we have assumed that 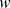 employs 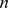 bits.
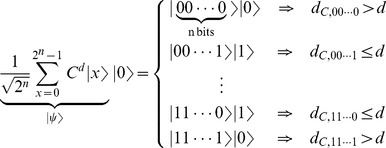
(1)


More generally, suppose that the compound system after the unitary evolution 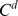 is in the entangled state represented by the right-hand side of Expression 2. Also, assume that the probability of observing the halting qubit 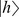 with outcome 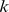 is 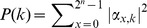. The projection postulate implies that we obtain a post observation state of the whole system as the one illustrated in Expression 3, where the system is projected to the subspace of the halting register and renormalized to the unit length [7].
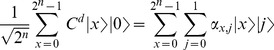
(2)

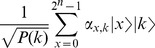
(3)


Consequently, observing the halt qubit after 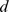 computational steps have been applied, will result in the working register containing either: (1) a superposition of the non-terminating states; or (2) a superposition of the halting states. Such behaviour has the to dramatically disturb a computation since: (1) a halting state may not always be obtained upon measurement due to random collapse, if indeed there exists one; and (2) any computation performed subsequently using the contents of the working register 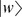 may employ an adulterated superposition with direct consequences on the interference pattern employed. Roughly speaking, there is no way to know whether the computation is terminated or not without measuring the state of the machine, but, on the other hand, such a measurement may dramatically disturb the current computation.

### Current approaches to the quantum halting problem

Ideally, one could argue that any von Neumann measurement should only be performed after all parallel computations have terminated. Indeed, some problems may allow one to determine 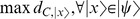, *i.e.* an upper-bound 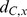 on the number of steps required for every possible input 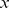 present in the superposition. However, this procedure is not viable for those problems which, like the *Entscheidungsproblem*, are undecidable. Bernstein and Vazirani subsequently proposed a model for a universal quantum Turing machine in [8] which did not incorporate into its definition the concept on non-termination. Although their model is still an important theoretical contribution it is nonetheless only capable of dealing with computational processes whose different branches halt simultaneously or fail to halt at all. These same arguments were later employed by Myers in [9] who argues that it is not possible to precisely determine for all functions that are Turing-computable, respectively 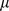-recursive functions, the number of computational steps required for completion. Additionally, the author also states that the models presented in [6] and [8] cannot be qualified as being truly universal since they do not allow for non-terminating computation. The work described in [8] is also restricted to the class of quantum Turing machines whose computational paths are synchronized, *i.e.* every computational path is synchronized in the sense that they must each reach an halt state at the same time step. This enabled the authors to sidestep the halting problem.

Following Myers observation of the conflict between quantum computation and system observation a number of authors provided meaningful contributions to the question of halting in quantum Turing machines. Ozawa [10]–[11] proposed a possible solution based on quantum nondemolition measurements, a concept previously employed for gravitational wave detection. Linden [12] argued that the standard halting scheme for Turing machines employed by Ozawa is unitary only for non-halting computations. Additionally, the author described how to build a quantum computer, through the introduction of an auxiliary ancilla bit that enabled system monitoring without spoiling the computation. However, such a scheme introduced difficulties regarding different halting times for different branches of computation. These restrictions essentially rendered the system classical since no useful interference occurred. In [13] expands the halting scheme described in [10] in order to introduce the notion of a well-behaved halting flag which is not modified upon completion. The author showed that the output probability distribution of monitored and non-monitored flags is the same. Miyadera proved that no algorithm exists capable of determining if an arbitrarily constructed quantum Turing machine halts at different computational branches [14]. Iriyama discusses halting through a generalized quantum Turing machine that is able to evolve through states in a non-unitary fashion [15].

Measurement-based quantum Turing machines as a model for computation were defined in [16] and [17]. Perdrix explores the halting issue by introducing classically-controlled quantum Turing machines [18], in which unitary transformations and quantum measurements are allowed, but restricts his model to quantum Turing machines that halt. Muller shows the existence of a universal quantum Turing machine that can simulate every other quantum Turing machine until the simulated model halts which then results in the universal machine halting with probability one [19], [20]. The author describes operators that do not disturb the computation as long as the original input employed halts the calculation process. This requires presenting a precise definition of the concept of halting state. This notion results in a restriction where large parts of the domain are discarded since the definition requirements are not met.

In [21] a method is presented for verifying the correctness of measurement-based quantum computation in the context of the one-way quantum computer described in [22]. This type of quantum computation differs from the traditional circuit based approach since one-qubit measurements are performed on an entangled resource labeled as a cluster state in order to mold a quantum logic circuit on the state. With each measurement the entanglement resource is further depleted. These results are further extended in [23] in order to prove the universality of the computational model. Subsequently, in [24] these concepts were used in order to prove that one-way quantum computations have the same computational power as quantum circuits with unbounded fan-out. Perdrix [25] discusses partial observation of quantum Turing machines which preserve the computational state through the introduction of a weaker form of the original requirements of linear and unitary 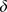 functions suggested by Deutsch in [6]. Recently, [26] proved that measurements performed on the 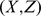-plane of the Bloch sphere over graph states is a universal measurement-based model of quantum computation.

### Objectives

In its seminal paper [6], Deutsch emphasizes that a quantum computer needs the ability to operate on an input that is a superposition of computational basis in order to be “fully quantum”, When confronted with the halting issue Myers naturally raised the question if a universal quantum computer could ever be fully quantum? And how would such a computational model eventually function? We aim to provide an answer to these questions by developing an alternative proposal to quantum Turing machines based on production system theory. We introduce such a computational model in order to gain additional insight into the matter of halting and universal computation from a different perspective than that of the standard quantum Turing machine.

As Miyadera stated, the notion of probabilistic halting in the context of quantum Turing machines cannot be avoided, suggesting that the standard halting scheme of traditional quantum computational models needs to be reexamined [14]. Our proposal is essentially different from the ones previously discussed since it imposes a strict notion of how the computation is performed and progresses in the form of the sequence of instructions that should be applied. Our method evaluates 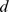-length sequences of instructions representing different branches of computation, enabling one to determine which branches, if they exist, terminate the computation. Underlying the proposed model will be Grover's algorithm in order to amplify the amplitude of potential halting states, if such states exist, and thus avoiding obtaining a random projection upon measurement. As a result, we will focus on characterizing the computational complexity associated with such a model and showing that it does not differ from that of Grover's algorithm.

With this work we are particularly interested in: (1) preserving the original principles proposed by Deutsch of linearity and unitary operators, in contrast with other proposals such as [25] and [15] which perform modifications to the underlying framework; (2) developing a model which considers all possible computational paths and (3) works independently of whether the computation terminates or not taking into account each possible computational path. Additionally, we will also consider some of the implications of being able to circumvent the halting problem. Computation universality is a characteristic attribute of several classical models of computation. For instance, the Turing machine model was shown to be equivalent in power to lambda calculus and production system theory. Accordingly, it would be interesting to determine what aspects of such a relationship are maintained in the context of quantum computation. Namely, we are interested in determining if it is possible to simulate a classical Turing machine given a quantum production system.

### Organisation

The rest of this introduction presents an overview of the main concepts required for a complete understanding of the results that will be presented, namely: (1) Subsection **“Production System Review”** presents the details of production system theory, a computational model that will be employed to model tree search applied to the halting problem; (2) Subsection “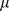 **-recursive functions”**presents a class of functions that contemplate non-terminating computation; and (3) Subsection **“Grover's Algorithm”** describes the quantum search algorithm. The remainder of this work is organised as follows: Section **“Results and Discussion”** extends the ideas presented in the introduction in order to: (1) elaborate on how Grover's algorithm can be extended in order to examine computational paths in Subsection **“Quantum Production System Oracle”**; and (2) discuss the details associated with our proposal for detection of quantum halting states in Subsection **“Quantum Iterative Deepening”**. Section **“Analysis”** then describes how such a method: (1) does not differ in complexity terms from the original Grover algorithm in Subsection **“Complexity Analysis”**; and (2) can be employed in order to coherently simulate a classical Turing machine in Subsection **“Turing machine simulation”**.

### Production System Review

Our approach to the detection of quantum halting states requires fixing a computational model. This step is required since our proposal depends on the set of state transitions occurring during a computational process. We choose not to focus on Turing machines, instead our proposal will be formulated in terms of production system theory. This decision is based on the fact that the quantum Turing machine model was already well explored by Deutsch [6] as well as Bernstein and Vazirani [8]. Furthermore, the combination of quantum concepts such as interference, entanglement and the superposition principle alongside the halting issue also contribute to make these models inherently complex. As a result, it is difficult to express elementary computational procedures. This behaviour contrasts with the simplicity of production system theory which allows for an elegant and compact representation of computations.

Production system theory is also well suited to support tree search, a form of graph search from which we drew our initial inspiration. In addition, the classical counterparts of both models were shown to be equivalent in computational power [27]. The production system is a formalism for describing the theory of computation proposed by Post in [28], consisting of a set of production rules 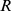, a control system 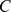 and a working memory 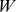. This sections reviews some of the most significant definitions that were proposed in [29], namely:

#### Definition 1

Let 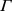 be a finite nonempty set whose elements are referred to as symbols. Additionally, let 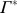 be the set of strings over 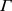.

#### Definition 2

The working memory 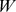 is capable of holding a string belonging to 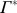. The working memory is initialized with a given string, who is also commonly referred to as the initial state 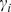.

#### Definition 3

The set of production rules 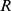 has the form presented in Expression 4.
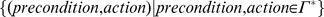
(4)


Each rules precondition is matched against the contents of the working memory. If the precondition is met then the action part of the rule can be applied, changing the contents of the working memory.

#### Definition 4

The tuple 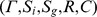 represents the formal definition of a production system where 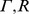 are finite nonempty sets and 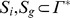 are, respectively, the finite sets of initial and goal states. The control function 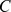 satisfies Expression 5.
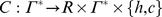
(5)


The control system 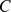 chooses which of the rules to apply and terminates the computation when a goal configuration, 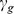, of the memory is reached. If 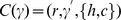 the interpretation is that, if the working memory contains string 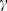 then it is substituted by the action 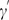 of rule 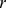 and the computation either continues, 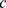, or halts, 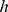. Traditionally, the computation halts when a goal state 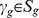 is achieved through a production, and continues otherwise.

#### Definition 5

Let 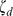 represent a sequence of productions leading up to a state 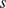 of length 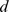. If 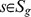 then such a sequence is also referred to as a solution.

**Figure 1:**
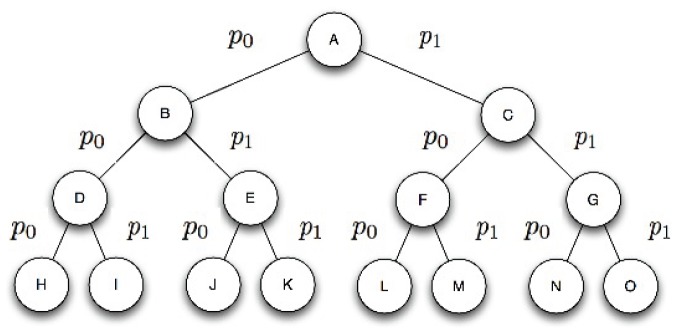
Tree structure representing the multiple computational paths of a probabilistic production system.

Figure 1 illustrates a production system with two production rules namely 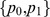 that can always be applied. Thus the representation as a graph with a tree form, representing a search of depth level 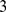 with initial state is 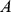 and leaf 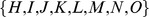. Each depth layer 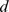 adds 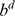 nodes to the tree, where 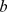 is the branching factor resulting from 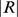, with each requiring a unique path leading to them. Therefore a total of 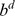 possible paths exist, *e.g.* state 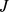 is achieved by applying sequence 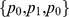.

With these definitions in mind it becomes possible to develop a suitable model for a quantum production system. Namely, the complex valued control strategy would need to behave as illustrated in Expression 6 where 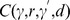 provides the amplitude if the working memory contains string 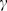 then rule 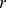 will be chosen, substituting string 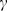 with 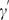 and a decision 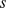 made on whether to continue or halt the computation.
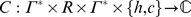
(6)


The amplitude value provided would also have to be in accordance with Expression 7, 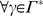


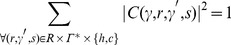
(7)


We will employ the notation described in [7] to describe the evolution of our quantum production system. Suppose we have a unitary operator 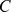 with the form presented in Expression 6. Operator 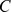 is responsible for a discrete state evolution taking the system from state 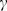 to 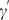 through production 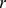, expressed as 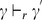. We refer to the transition 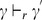 as a *computational step*. The computation of a production system starting in an initial state 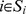 can be defined as a sequence of steps 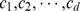 such that 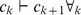 and where 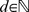 represents the depth at which a solution state 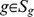 can be found. In general, the unitary operator 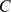 can be perceived as applying a single computational step of the control strategy for a general production system. This notation is convenient since we are able to express the computation of a production system 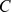 up to depth-level 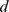 as 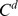, *i.e.* a depth-limited search mechanism that mimics the behaviour illustrated in Figure 1.

### 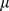-recursive functions

Universal models of computation are capable of calculating 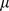-recursive functions, a class of functions which allow for the possibility of non-termination. These functions employ a form of unbounded minimalization, respectively the 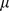-operator, which is defined in the following terms [3]: let 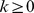, 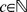,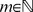 and 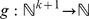, then the unbounded minimization of 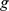 is function 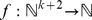 as illustrated in Expression 8, for any 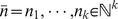.
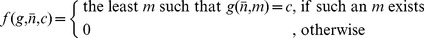
(8)


**Figure 2:**
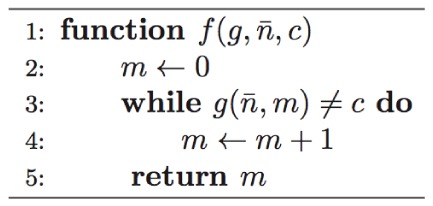
The classical 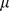
 **-operator (adapted from** [**30**] **).**

The unbounded minimization operator can be perceived as a computational procedure responsible for repeatedly evaluating a function with different inputs 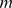 until a target condition 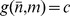 is obtained [30]. However, as illustrated by Expression 8, there is no guarantee that the target condition will ever be met. Accordingly, it is possible to express the inner-workings of 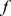 as an iterative search that may never terminate, as illustrated in Figure 2. Notice that although 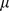-recursive functions employ a collections of variables belonging to the set of natural numbers, for practical purposes these values are restricted by architecture-specific limits on the number of bits available for representing the range of possible values.

From a quantum computation perspective, it is possible to perform a generic search for solution states through amplitude amplification schemes such as the one described by Grover in [31] and [32]. In this section we will discuss how to combine production system theory alongside the quantum search algorithm in order to develop a new computational model better suited to deal with the halting issue.

### Grover's algorithm

The quantum search algorithm employs an oracle 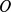 whose behaviour can be formulated as presented in Expression 9, where 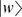 is a 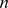-qubit query register, 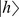 is a single qubit answer register. Additionally, 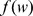 is responsible for checking if 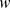 is a solution to a problem, outputting value 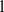 if so and 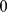 otherwise. In the context of this research we only consider deterministic functions.
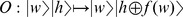
(9)


It is important to mention that we employed some care when defining the oracle in terms of registers 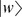 and 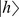, in a similar manner to the quantum Turing machine model proposed by Deutsch. We deliberately chose to do so in order to establish some of the connections between the halting problem and the quantum search procedure. We may view the halting problem as one where we wish to obtain the computational basis present in 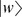 which lead to goal states 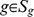 where 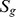 is defined as the set of halting states.

Grover's algorithm starts by setting up a superposition of 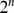 elements in register 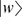 and subsequently employs a unitary operator 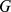 known as Grover's iterate [33] in order to amplify the amplitudes of the goal states and diminish those of non-goal states. The algorithm is capable of searching the superposition of 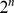 elements by invoking the oracle 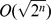 times. The computational complexity of 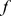 should also be taken into consideration. Namely, assume that 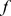 takes time 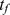. Since Grover's algorithm performs 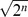 oracle invocations then the total complexity will be 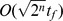. This complexity still represents a speedup over an equivalent classical procedure since 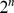 states would have to be evaluated independently. However, for a polynomial 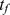 the overall complexity will be dominated by the dimension of the search space, *i.e.* 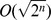. For this reason, it is often assumed that 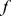 is computable in polynomial time. This assumption also makes such oracle models suitable to the complexity class NP which represents the class of languages that can be verified by a polynomial-time algorithm.

In addition it is also possible that the space includes several solutions. Accordingly, let 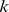 represent the number of solutions that exist in the search space, then the complexity of the quantum search algorithm can be restated as 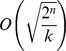. Typically, 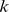 can be determined through the quantum counting algorithm described in [34] which also requires a similar time complexity. This means that before applying Grover's algorithm one must first determine the number of solutions. Overall, the time complexity of applying both methods sequentially remains the same. Once the algorithm terminates and a measurement is performed then a random collapse occurs, with high probability, amongst the amplified solutions. In the remainder of this work we gain generality by thinking in terms of the worst-case scenario where a single solution exists. However, the method described above could still be applied to the proposition that is described in the following sections. Grover's algorithm was experimentally demonstrated in [35].

## Results and Discussion

### Quantum Production System Oracle

Is it possible to present an adequate mapping of our quantum production system that is suitable to be applied alongside Grover's algorithm? A comparison of Expression 6 and Expression 9 allows us to reach the conclusion that oracle 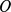 performs a verification whilst 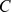 focuses on executing an adequate state evolution. Therefore, we need to develop an alternate mechanism that behaves as if performing a verification. We can do so by focusing on one of the main objectives of production system theory, namely that of determining the sequence of production rules leading up to a goal state. Formally, we are interested in establishing if an initial state 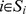 alongside a sequence of 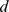 productions rules 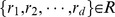 leads to a goal state 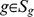. If the sequence of rules leads to a goal state, then the computation is marked as being in a halt state 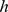, otherwise it is flagged to continue 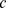. We can therefore proceed with a redefinition of the control function presented in Expression 6, as illustrated in Expression 10, which closely follows the oracle definition presented in Expression 9.
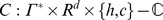
(10)


Recall that the oracle operator is applied to register 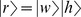. We choose to represent register 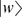 as a tensor of two products, namely 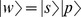, where 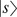 is responsible for holding the binary representation of the initial state and 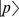 contains the sequence of productions. Register 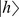 is utilized in order to store the status 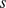 of the computation. Additionally, the revised version of the quantum production system 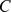 with oracle properties should also maintain a unit-norm, as depicted by Expression 11, 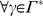. For specific details surrounding the construction of such a unitary operator please refer to [36].
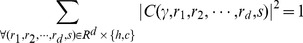
(11)


Any computational procedure can be described in production system theory by specifying an appropriate set of production rules that are responsible for performing an adequate state evolution. This set of production rules can be applied in conjunctions with a unitary operator 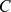 incorporating the behaviour mentioned in Expression 10 and Expression 11. In doing so we are able to obtain a derivation of a production system that can be combined with Grover's algorithm. From a practical perspective, we are able to initialize 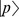 as a superposition over a set 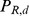 representing the sequence of all possible production rules 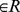 up to a depth-level 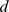, as illustrated by Expression 12 and Expression 13. Implicit to these definitions is the assumption that set 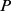 has a total of 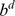 possible paths.
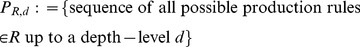
(12)

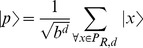
(13)


Traditionally, throughout a computation set 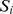 remains static in the sense that it does not grow in size. However, variable 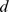 is constantly increased in order to generate search spaces covering a larger number of states. As a result, given a sufficiently large depth value the number of bits required for 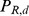 will eventually surpass the amount of bits required to encode set 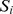. Accordingly, in the reasonable scenario where the number of bits required to encode the sequence of productions over 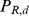 is much larger than the number of bits required to encode the set of initial states 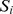, *i.e.* 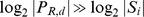, then the most important factor to the dimension of the search space will be the number of productions. For this reason, Grover's algorithm needs to evaluate a search space spanning roughly a total of 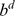 paths. As a consequence, the algorithm's running time is 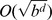 which effectively cuts the search depth in half [37].

### Quantum Iterative Deepening

Any approach to a universal model of quantum computation needs to focus on two main issues, namely: (1) how to circumvent the halting problem and (2) how to handle computations that do not terminate without disturbing the result of the procedure. In the next sections we describe our general procedure. We choose to focus first on the second requirement given that it provides a basis for model development by establishing the parallels between 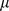-theory and production system theory. We then describe how these arguments can be utilized in order to develop a computational model capable of calculating 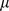-recursive functions.

#### Parallels between 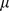-theory and production system theory

Universal computation must allow for the possibility of non-termination, a characteristic that is is achievable through the ability to calculate 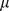-recursive functions. Therefore, the question naturally arises if it is possible to develop a quantum analogue of the iterative 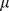-operator? By itself 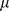-recursive functions are not seen as a model of computation, but represent a class of functions that can be calculated by computational models. Accordingly, we are interested in determining if we are able to develop a quantum computational model, namely by employing the principles of production system theory, capable of calculating 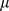-recursive functions without affecting the end result.

In order to answer this question we will first start by establishing some parallels between these concepts. Namely, consider the 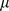-operator presented in Figure 2 that receives as an argument a tuple 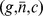 and a production system defined by the tuple 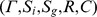. Accordingly, parameter 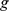 can be perceived as a control strategy 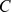 responsible for mapping a set of symbols 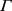 in accordance with a set of rules 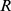. Variable 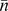 can be interpreted as an element of the set of initial states, *i.e.* 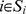. The target condition 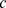 can be understood as the set of goal states 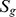. In addition, the unbounded minimization operator employs a parameter 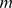 that represents the first argument where the target condition is met. Analogously, from a production system perspective, variable 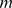 can be viewed as the first depth 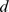 where a solution to the problem can be found. Finally, the condition 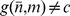 of the while loop is equivalent to applying the control strategy 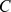 at total of 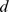 times, *i.e.* 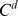, and evaluating if a goal state was reached.

#### Iterative Search

The fact that we are able to perform such mappings hints at the possibility of being able to develop our own quantum equivalent of the 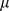-operator based on production system fundamentals. All that is required is a while loop structure, mimicking the iterative behaviour of the 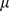-operator, that exhaustively examines every possibility for 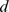 alongside 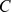, until a goal state is found. Since we need to evaluate if applying 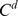 leads to a solution we can combine the quantum production system oracle presented in Expression 10 alongside Grover's iterate for a total of 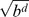 times in order to evaluate a superposition of all the available sequences of productions up to depth-level 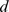, *i.e.* 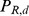. After applying Grover's algorithm, we can perform a measurement 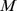 on the superposition, if the state 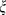 obtained is a goal state, then the computation can terminate since a solution was found at depth 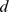.

**Figure 3:**
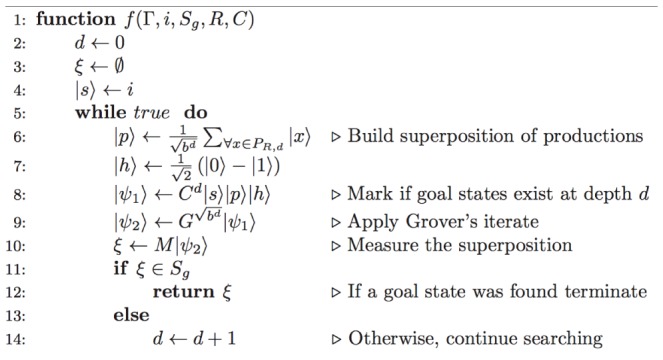
The quantum iterative deepening procedure.

This process is illustrated in Figure 3 which receives as an argument a tuple 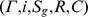, where 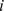 is an initial state, *i.e.* 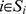. We choose to represent our procedure as a form of pseudocode that is in accordance with the conventions utilized in [38], namely: (1) indentation indicates block structure, *e.g.* the set of instructions of the while loop that begins on line 5 consists of lines 6 - 14; (2) we use the symbol 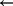 to represent an assignment of a variable; and (3) the symbol 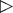 indicates that the remainder of the line is a comment.

Line 8 is responsible for applying the oracle alongside an initial state and all possible sequences of productions. Recall that register 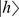 will be set if goal states can be reached. Line 9 is responsible for applying Grover's algorithm. If goal states are present in the superposition, then Grover's amplitude amplification scheme allows for one of them to be obtained with probability 
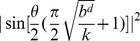
 [39], where 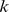 represents the number of solutions and 
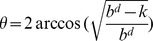
. It is possible that state 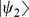 contains a superposition of solutions. Therefore, measuring the system in Line 10 will result in a random collapse amongst these. If the measurement returns an halt state, then register 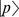 will contain a sequence of productions leading to a goal state. Once the associated sequence has been obtained one has only to apply each production of the sequence in order to determine precisely what was the goal state obtained [36] (Line 11). Otherwise, the search needs to be expanded to depth level 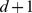 and the production evaluation process repeated from the start. As a result, this procedure requires building a new superposition of productions 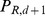 each time a solution was not found in 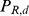.

Due to the probabilistic nature of Grover's algorithm there is also the possibility that the measurement will return a non halting state, even though 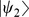 might have contained sequences of productions that led to goal states. This issue can be circumvented to a certain degree. Notice that the sequences expressed by 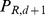 also contain the paths 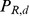 as subsequences. This means that when 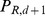 is evaluated the iteration procedure has the opportunity to re-examine 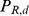. As a result, operator 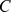 would have the chance to come across the exact subsequences that had previously led to goal states but that were not obtained after the measurement. Therefore, the control strategy would need to be modified in order to signal an halt state as soon as a solution is found, *i.e.* the shallowest production, independently of the sequence length being analyzed. With such a strategy the probability of obtaining a non-halting state in each unsought iteration level 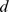 would be 
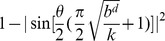
.

Each iteration of Figure 3 starts by building a superposition 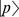 spanning the respective depth level. This means that the original interference pattern that was possibly lost upon measuring the system in the previous iteration is rebuilt and properly extended by the tensor product that is performed with the new productions. Because of this process the computation is able to proceed as if undisturbed by the measurement. Such a reexamination comes at a computational cost which will be shown to be neglectable. This behaviour contrasts with the original approach discussed by Deutsch where: (1) a computation would be applied to a superposition 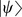; (2) a measurement would eventually be made on the halt qubit collapsing the system to 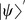 and (3) if a goal state had not been obtained the computation would proceed with 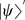.

## Analysis

### Complexity Analysis

Figure 3 represents a form of iterative deepening search, a general strategy employed alongside tree search, that makes it possible to determine an appropriate depth limit 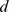, if one exists [40]. The first documented use of iterative deepening in the literature is in Slate and Atkin's Chess 4.5 program [41], a classic application of an artificial intelligence problem. Notice that up until this moment we had not specified how to obtain a value for depth 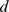, this was done deliberately since the essence of 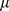-recursive functions relies in the fact that such a value may not exist. In general, iterative deepening is the preferred strategy when the depth of the solution is not known [40]. Accordingly, the while loop will execute forever unless the state 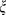 in line 11, obtained after the measurement, is a goal state.

Since we employ Grover's algorithm we do not need to measure specifically the halting register. Instead it is possible to perform a measurement on the entire Hilbert space of the system in order to verify if a final state is obtained. This type of a control structure is responsible for guaranteeing the same type of partial behaviour that can be found on the classical 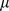-operator. Consequently, Figure 3 also does not guarantee that variable 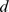 will ever be found, *i.e.* the search may not terminate. Line 8 of the algorithm uses the register 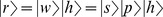 previously described.

Quantum iterative deepening search may seem inefficient, because each time we apply 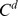 to a superposition spanning 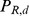 we are necessarily evaluating the states belonging to previous depth levels multiple times, 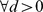. However, the bulk of the computational effort comes from the dimension of the search space to consider, respectively 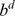, which grows exponentially fast. As pointed out in [42] if the branching factor of a search tree remains relatively constant then the majority of the nodes will be in the bottom level. This is a consequence of each additional level of depth adding an exponentially greater number of nodes. As a result, the impact on performance of having to search multiple times the upper levels is minimal. This argument can be stated algebraically by analysing the individual time complexities associated with each application of Grover's algorithm for the various depth levels. Such a procedure is illustrated in Expression 14 which gives an overall time complexity of 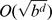 remaining essentially unchanged from that of the original quantum search algorithm.
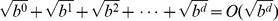
(14)


By employing our proposal we are able to develop a quantum computational model with an inherent speedup relatively to its classical counterparts. Notice that this speedup is only obtained when searching through a search space with a branching factor of at least 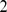 (please refer to [37] [36]). In addition, if the set of goal states is defined to be the set of halt states, then we are able to use our algorithm to circumvent the halting problem. Our method is able to do so since it can compute a result without the associated disruptions of Deutsch's model. We employ such a term carefully, since it may be argued that the measurements performed during computation will inherently disturb the superposition. This is not a problem if a halt state is found. However, if such a goal state is not discovered, we move on to an extended superposition through 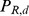, representing an exponentially greater search space, where the states from the previous tree levels are included. Consequently, it becomes possible to recalculate the computation as if it had not been disturbed and without changing the overall complexity of the procedure.

### Turing machine simulation

The approach proposed in this work allows for the possibility of non-termination, without inherently interfering with the results of the quantum computation. This hints at the possibility that our approach can be applied to coherently simulate classical universal models of computation such as the Turing machine. Specifically, we are interested in determining what would be needed for our model of an iterative quantum production system to simulate any classical Turing machine?

We will begin by presenting a set of mappings between Turing machine concepts and production system concepts in a manner analogous to the trivial mapping described in [43]. Both models employ some form of memory where the current status of the computation is stored. The Turing machine model utilises a tape capable of holding symbols. Each element of the tape can be referred to through a location. Tape elements are initially configured in a blank status, but their contents can be accessed and modified through primitive read and write operations. These operations are performed by a head that is able to address each element of the tape. As a result, the memory equivalent of the production system, respectively, the working memory should convey information regarding the current head position and the symbols, alongside the respective locations, on the tape. In addition, the tape employed in Turing's model has an infinite dimension. Consequently, the working memory must also possess an infinite character.

The Turing machine model utilises a 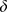 function to represent finite-state transitions. The 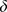 functions maps an argument tuple containing the current state and the input being read to tuples representing a state transition, an associated output and some type of head movement. This set of transitions can be represented as a table whose rows correspond to some state and where each column represents some input symbol. Each table entry contains the associated transition tuple representing the next internal state, a symbol to be written, and a head movement. Notice, that this behaviour fits nicely into the fixed set of rules 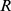 employed by production systems. Namely, 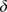
's argument and transition tuples can be seen, respectively, as a precondition and associated action of a certain rule. Accordingly, for each table entry of the original Turing transition function it is possible to derive an adequate production rule, thus enabling the obtention of 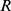.

The only remaining issue resides in defining a control strategy 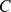 that mimics the behaviour presented in Expression 10. Consequently 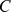 needs to choose which of the rules to apply by accessing the working memory, determining the element that is currently being scanned by the head, and establishing if a goal state is reached after applying some specific sequence of 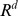 rules. Once this is done, we are able to apply our iterative quantum production system to simulate the behaviour of a classical Turing machine. The 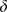-function conversion to an adequate database of productions is a simple polynomial-time procedure (please refer to [27] and [44] for additional details). In addition, it is important to mention that this approach will only provide a speedup if the Turing machine simulated allows for multiple computational branches. Otherwise, if the computation is not capable of being parallelized then we gain nothing, performance-wise, from employing quantum computation.

## Conclusions

In this work we presented an approach for an iterative quantum production system with a built-in speedup mechanism and capable of the partial behaviour characteristic of 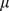-recursive functions. Our proposal makes use of a unitary operator 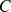 that can be perceived as mapping a total function since it maps for every possible input into a distinct output. However, operator 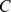 is employed in a quantum iterative deepening procedure that examines all path possibilities up to a depth level 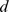 until a solution is found, if indeed there exists one. Due to the probabilistic nature of Grover's algorithm there is always the possibility that, upon measurement, a non-terminating state is obtained. As a consequence, the procedure would iterate to an additional level of productions and could therefore fail to recognize a halting state. This issue can be overcome through the development of specific control strategies capable of signaling that an halting state has been found at the shallowest production yielding such a conclusion, independently of the sequence length being analyzed.

Our model is able to operate independently of whether the computation terminates or not, a requirement associated with universal models of computation. As a result, it becomes possible for our model to exhibit partial behaviour that does not disturb the overall result of the underlying quantum computational process. This result is possible since: (1) Grover's algorithm effectively allows one to obtain halting states, if they exist, with high probability upon system observation; and (2) the overall complexity of this proposition remains the same of the quantum search algorithm. This procedure enables the development of verification-based universal quantum computational models, which are capable of coherently simulating classical models of universal computation such as the Turing machine.
